# Do threats and reassurances reside in the biological, psychological or social domain? A qualitative study in adults and young people with chronic pain

**DOI:** 10.1177/20494637241263291

**Published:** 2024-06-24

**Authors:** Hannah Kennedy, Daniel S Harvie, Michel W Coppieters

**Affiliations:** 1 School of Health Sciences and Social Work, Griffith University, Brisbane and Gold Coast, QLD, Australia; 2 Interdisciplinary Persistent Pain Centre, 3556 Gold Coast Hospital and Health Service, Gold Coast, QLD, Australia; 3 Innovation, Implementation and Clinical Translation in Health (IIMPACT in Health), Allied Health and Human Performance, 1067 University of South Australia, Adelaide, SA, Australia; 4 Amsterdam Movement Sciences, Program Musculoskeletal Health, Faculty of Behavioural and Movement Sciences, 1190 Vrije Universiteit Amsterdam, Amsterdam, The Netherlands

**Keywords:** Biopsychosocial, pain management, education, children

## Abstract

**Objective:**

Understanding biopsychosocial contributions to a sensitised pain system is a key target of many pain management programs. The ‘Protectometer’ is a freely available educational tool that guides people with chronic pain to explore their personal threats and reassurances, identifying them as ‘DIMs’ (danger in me) or ‘SIMs’ (safety in me), to guide personalised pain management. This study aimed to explore common types of DIMs and SIMs, and examine differences between adults and young people.

**Materials and Methods:**

A retrospective qualitative study was conducted. Written DIMs (*n* = 504) and SIMs (*n* = 711) were collected from 96 participants with chronic pain (77 adults aged 18–85 years; 19 young people aged 9–17 years) across 15 multidisciplinary pain management groups. DIMs and SIMs were transcribed and analysed using deductive content analysis.

**Results:**

Four overarching themes were identified: ‘Engaging with the environment’, ‘In my body’, ‘My emotional health’, and ‘Activities and behaviours’. Similarities in SIMs were found, with the greatest proportion of SIMs in the social domain (49% adults; 47% young people). While adult DIMs were fairly evenly spread across the biological (37%), psychological (27%) and social domains (36%), young people’s DIMs were predominantly in the psychological (44%) and social (43%) domains.

**Discussion:**

These findings provide insights into common threats and reassurances people in pain perceive, and revealed age-related differences in biopsychosocial contributions to pain and pain relief. Findings also highlight the importance of social-based interventions as part of pain management therapies for both adults and young people.

## Introduction

Developing an understanding of pain is a key component of chronic pain management. By understanding more about the factors contributing to ongoing pain, people with pain have demonstrated greater participation in treatment, less catastrophic beliefs, and reductions in pain intensity.^1–3^ However, ‘understanding pain’ requires more than a didactive biology-focussed knowledge transfer approach. Rather, it requires engagement of the learner in a process of conceptual change regarding the meaning of pain and its biopsychosocial contributors.^4,5^

Taking into account that educational strategies are only effective to the extent that people in pain adopt new concepts and behaviours,^6^ it is beneficial that clinicians have a range of tools to provide pain education. Individualised and meaningful educational approaches have shown improved retention and engagement across many areas of healthcare.^7,8^ The ‘Protectometer’ is one such approach, a freely available tool designed to increase patients’ insight in the factors that influence their ongoing pain state.^9^ People in pain are guided to explore personal factors which they perceive to either increase or decrease their pain experience. These are identified as ‘DIMs’ (danger in me) which are said to amplify protective systems, including pain, or ‘SIMs’ (safety in me), which are said to suppress protective systems, including pain. The aim of the ‘Protectometer’ is to explore personal DIMs and SIMs, modify and/or remove DIMs as much as possible, and gather and/or increase attention towards SIMs, with the goal to temper the pain system towards a less sensitive state.^9^ The lack of data on types of DIMs and SIMs in people with pain is an important gap in our current understanding and use of the ‘Protectometer’ in pain management.

The ‘Protectometer’ encourages a broad exploration of the context involved in ongoing pain, through seven categories of potential DIMs and SIMs: (1) things you hear, see, smell, taste, touch; (2) things you do; (3) things you say; (4) things you think and believe; (5) places you go; (6) people in your life; and (7) things happening in your body.^9^ In theory, this holistic approach to understanding and treating pain reflects the biopsychosocial model, however, there has been no evaluation of whether the types of DIMs and SIMs generated in practice actually align with this model. Additionally, while a biopsychosocial approach is recommended for pain management in both young people and adults with chronic pain,^10,11^ the nature of DIMs and SIMs expressed at different ages has not been explored.

Therefore, the aims of this study were to: (1) explore types of DIMs and SIMs identified by people using the ‘Protectometer’ in multidisciplinary pain management group programs, (2) examine how DIMs and SIMs are distributed across domains of the biopsychosocial model, and (3) explore differences and similarities in DIMs and SIMs between young people and adults.

## Materials and Methods

### Study design

This multi-site study used a qualitative design to understand the types of threats and reassurances elicited by people using the ‘Protectometer’ during pain management group programs. Ethical approval for this study was obtained from the Human Research Ethics Committee at the relevant health service (HREC/2021/QGC/80034) and university (GU Ref No: 2021/829). A waiver of consent of the participants was approved, as data were collected retrospectively in a de-identified format.

### Study sites

Data were retrospectively collected from two study sites: the Gold Coast Interdisciplinary Persistent Pain Centre (IPPC), a tertiary-referral, public pain centre for people with chronic pain over the age of 18 years, and Support Kids in Pain (SKiP), a non-governmental organisation, primary care pain centre for people with chronic pain under the age of 18 years. Both sites facilitated an outpatient, 4-week multidisciplinary pain management program, in line with evidence-based guidelines.^12^ The IPPC adult program comprised of 8 five-hour sessions (i.e. 2 days a week for 4 weeks) and the SKiP young person program comprised of 4 six-hour sessions (i.e. 1 day a week for 4 weeks). Both sites used the ‘Protectometer’ as a component of the pain education sessions and had stored DIMs and SIMs in a manner suitable for collection and analysis.

### Participants

Participants were people with chronic non-cancer pain (lasting longer than 3 months) who were enrolled in pain management programs at either study site between 2019 and 2021. Fifteen pain management groups were conducted during the study period: 10 at the adult site and 5 at the young person site, with a total of 96 participants (adults *n* = 77, young people *n* = 19). The mean (SD) age of the young people group was 14 (2) years, with a majority of female participants (15/19). The adult group mean (SD) age was 56 (15) years, also with a majority of female participants (50/77). Demographic characteristics of participants are displayed in Table 1.

**Table 1.** Demographic characteristics of the adult and young people groups.

	Adults (*n* = 77)	Young people (*n* = 19)
Age	18–34 years: 8 (10%)	9–12 years: 8 (42%)
	35–54 years: 22 (29%)	13–17 years: 11 (58%)
	55–74 years: 42 (54%)	
	75–85 years: 5 (7%)	
Sex	Female: 50 (65%)	Female: 15 (79%)
	Male: 27 (35%)	Male: 4 (21%)
Pain duration^a^	<1 year: 1 (1%)	<1 year: 7 (37%)
	1–3 years: 16 (21%)	>1 year: 12 (63%)
	3–5 years: 20 (26)	
	5–10 years: 12 (16%)	
	>10 years: 28 (36%)	

^a^Different pain duration classifications were used at the participating sites.

### The ‘Protectometer’

The ‘Protectometer’ was used at both sites as part of their multidisciplinary pain management group programs. At the adult site, the ‘Protectometer’ was introduced in week two of the 4-week pain management program. In a one-hour session, the concept of pain as a protective system was explored, and participants brainstormed their own DIMs in each of the tool’s seven categories. Participants wrote their DIMs onto sticky notes and placed them on a large group poster. Over the following 2 weeks, participants gradually added SIMs to the poster through a ‘SIM hunt’ and other SIM finding activities, with the group goal to have more SIMs than DIMs by the end of the program. At the young person site, the ‘Protectometer’ was also introduced in week two of their 4-week pain management program, and was completed in a single 45-min session. In this session, both DIMs and SIMs were explored, written on sticky notes by participants, and placed on a large group poster that was displayed for the remainder of the program. A mixed roster of allied health staff (occupational therapists, physiotherapists and psychologists) facilitated the group programs.

### Data collection

The sticky notes with DIMs and SIMs were collected at the completion of each program, as well as demographic details of each group (age, gender and pain duration). A total of 1215 DIMs and SIMs were collected, with 383 DIMs and 516 SIMs at the adult site, and 121 DIMs and 195 SIMs at the young person site. Data from the sticky notes were entered by the primary researcher (HK) into Microsoft Excel in groups of adult DIMs, adult SIMs, young person DIMs and young person SIMs.

### Qualitative analysis

Text extracted from the sticky notes were analysed using deductive content analysis to allow a structured examination of written data and their meanings.^13^ Qualitative content analysis provides subjective interpretation of the content of text, through a systematic classification process of coding and identifying themes.^14^ Content analysis also allows systematic counting to quantify differences between age groups.^15^

Themes were identified through a deductive approach, where coding was based on previous knowledge^16^ of core domains in the biopsychosocial model of pain.^10,11,17,18^ Firstly, the primary researcher (HK) became familiar with the data through transcription and repeated readings of the data. Together with a second researcher (DH), data were arranged into groups, which were then coded and named (categories).^19^ The categories were then grouped into larger themes, and a codebook was drafted.

A semantic approach was used where codes and potential themes were identified at the surface level of what participants had written, rather than interpreting underlying meanings or assumptions (latent approach).^20^ Furthermore, a peer debriefing process occurred with the third researcher (MC) to reduce the risk of bias. Fortnightly meetings were held during coding development and throughout the coding phase, to assist decision-making for any DIMs or SIMs that could fit several categories or had many components. In the instance that a DIM or SIM listed more than one component, primary coding rules were set and agreed upon within the team. For example, ‘colouring with kids’ was coded as a ‘positive social environment’, due to the inclusion of the additional information ‘with kids’, rather than being coded as ‘enjoyable activity’. Although the research team noted how the themes generated by deductive content analysis could be mapped onto the biopsychosocial model, the decision was made to generate themes independent of the biopsychosocial model terminology.

To measure distribution across domains of the biopsychosocial model, DIMs and SIMs were again analysed using a deductive content analysis into a pre-existing coding framework of biological, psychological or social domains. The coding framework was based on contemporary literature on the biopsychosocial model of pain across the lifespan,^10,11,17,18^ and agreed upon by the research team prior to coding. To quantify frequencies and comparison between age groups, DIMs and SIMs categories were ranked from most common to least common using percentages. Counts of DIMs and SIMs in the biological, psychological and social domains were totalled and converted to percentages for each domain.

Finally, we sought input from two people with lived experience of chronic pain, who were not involved in the pain programs, to (1) review and suggest any changes to recommended coding, and (2) provide feedback on theme names to reflect the intent of the themes. The research team consisted of clinicians and researchers with backgrounds in Occupational Therapy (HK) and Physiotherapy (DH & MC). The team members have a deep understanding of pain and pain management, as clinicians working in chronic pain management, and as educators, authors and researchers. They are experienced in qualitative research in the area of pain [e.g. References 21–24], while the two people with lived experience of chronic pain are both involved in peer support roles for people with chronic pain.

## Results

### Themes

Four themes were generated from deductive content analysis of the data, reflecting the main types of DIMs and SIMs: (1) Engaging with the environment, (2) In my body, (3) My emotional health and (4) Activities and behaviours. Within these themes, 15 DIM categories and 14 SIM categories were generated. The themes and categories are listed in Table 2. The two people with chronic pain supported the chosen themes and categories names and recommended three minor changes, which were implemented.

**Table 2.** Themes and categories from deductive content analysis of DIMs and SIMs.

Theme	DIMs categories	SIMs categories
Engaging with the environment	Threatening places	Comforting places
	Negative social environments (family, peers, work, school)	Positive social environments (family, peers, work, school)
	Negative healthcare environments	Positive healthcare environments
	Not being understood by people	Being understood by people
In my body	Health concerns	Health reassurances
	Negative sensory experiences	Positive sensory experiences
My emotional health	Negative self-talk and beliefs	Positive self-talk and beliefs
	Negative recovery expectations	Positive recovery expectations
	Low emotions	Pleasant emotions
	Worry and catastrophising	Positive spiritual beliefs
	Frustration and anger	
	Perceived injustice	
	Financial stress	
Activities and behaviours	Activity limitations and triggers	Active self-management skills
	Withdrawal	Enjoyable activities
		Making progress
		Meaningful contributions

#### Theme 1: Engaging with the environment

This theme described DIMs and SIMs from a variety of physical, social and cultural environments. There were four categories. Physical environments were captured in the category Places (threatening or comforting), and included places such as ‘shopping centres’, ‘the beach’ and ‘garden’; the Social environments category (positive or negative) included family, peers, school and work groups (e.g. ‘boss’, ‘wife’, ‘husband’, ‘friends’); Healthcare environments (positive or negative) included both interactions with healthcare professionals, and healthcare-related environments, such as ‘my physio’ and ‘hospital’. (Not) being understood by people included statements such as ‘people just don’t get it – I am so misunderstood’, and ‘I can talk to a teacher at school who understands’.

#### Theme 2: In my body

This theme encompassed sensory inputs and health-related experiences, with two categories. The Health concerns or reassurances category captured DIMs such as ‘I am so tired’ and ‘I hate all the medication I have to take’, and SIMs such as ‘changing how I take medication’ and ‘explaining the condition’. The Sensory experiences (positive or negative) category described DIMs such as ‘headaches’, ‘cramping’, and ‘stiffness’, and SIMs such as ‘a warm bath’, ‘deeply inhale frangipani scent’ and ‘listening to music’.

#### Theme 3: My emotional health

This theme related to both affective states and emotional responses. It comprised of the most categories, reflecting clusters of emotion-based DIMs or SIMs, for example, ‘get mad at my pain’ and ‘out of control’ were classed in the Frustration and anger category, while ‘I worry about everything’ and ‘anything I do will cause a dislocation’ were in the Worry and catastrophising category. SIM subthemes included Positive self-talk and beliefs (e.g. ‘I can do this’), Pleasant emotions (e.g. ‘positivity’, ‘happiness’) and Positive recovery expectations (e.g. ‘the pain experience can change’). See Table 2 for all categories.

#### Theme 4: Activities and behaviours

This theme related to DIMs or SIMs for activities, tasks and behavioural responses. DIM categories were Activity limitations and triggers, for example, ‘pushing a shopping trolley’, ‘going for a long drive without stopping’ and Withdrawal, such as ‘staying at home in the dark’. There were four SIM categories: Active self-management skills such as ‘exercise daily’ and ‘mindful breathing’, Enjoyable activities which included ‘thrift shopping’ and ‘making soaps’, Making progress (e.g. ‘seeing small improvements’) and Meaningful contributions (e.g. ‘Scouts make me feel useful, like a contributing member of society’).

### Biopsychosocial domains of DIMs and SIMs

**Figure 1.**
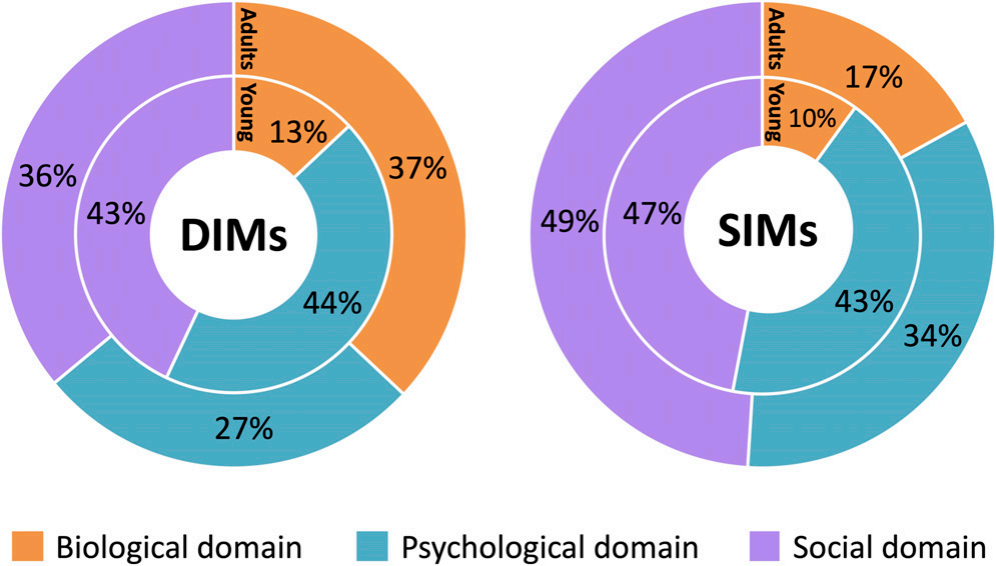
Proportions of DIMs and SIMs in biological, psychological and social domains for adults (outer ring) and young people (inner ring).

Adult DIMs were spread fairly evenly between domains of the biopsychosocial model, with 37% in the biological domain, 27% in the psychological domain, and 36% in the social domain (see Figure 1). ‘Clicking and clacking noises of neck and back’ and ‘lifting anything heavy’ were examples of participant DIMs coded into the biological domain; while ‘being out in public’ and ‘everyone who doesn’t understand’ were coded to the social domain; and ‘It’s not worth it’ and ‘I’m overwhelmed’ were coded into the psychological domain. Young people had most DIMs (44%) in the psychological domain (e.g. ‘I’ll never get better’), closely followed by the social domain (43%) (e.g. ‘friends don’t understand’), and the least (13%) in the biological domain (e.g. ‘I eat less’).

SIMs between the two groups were much more similar with respect to their distribution across the biopsychosocial model (Figure 1). Both adults and young people had the highest portion of SIMs in the social domain (49% and 47%, respectively), with SIMs such as ‘cooking something that all the family loves’, ‘the beach’, ‘my son takes me out in my wheelchair and we have lunch’ and ‘going to school to play with my friends’. The psychological domain had the second highest number of SIMs for both adults and young people (34% and 43%, respectively) (e.g. ‘focus on the good things around you’, ‘I believe in myself’), with the smallest number of SIMs in the biological domain (adults: 17% and young people: 10%) (e.g. ‘have a rest’).

### Key differences between adults and young people

**Figure 2.**
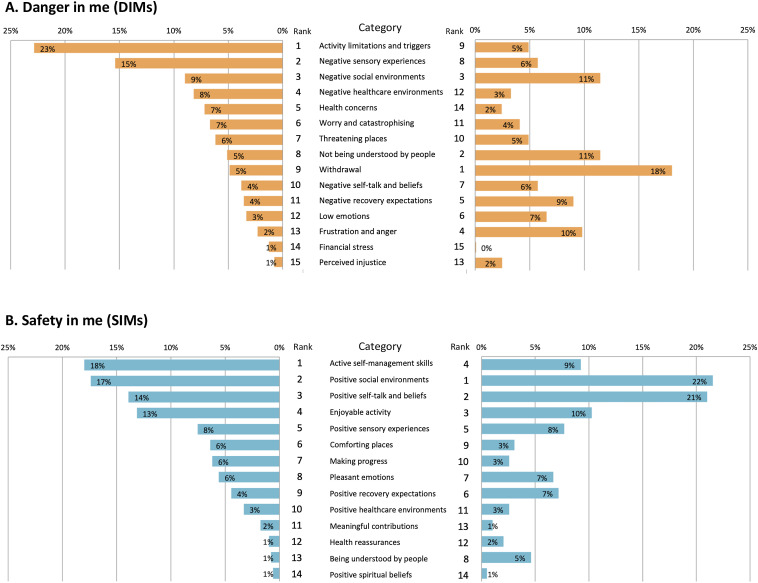
Frequency and rank of DIMs (Panel A) and SIMs (Panel B) categories for adults (left) and young people (right). Categories are ordered by descending frequency for adults.

Differences were seen in the most common types of DIMs between adults and young people. The top four most common DIM categories for adults were: Activity limitations and triggers, Negative sensory experiences, Negative social environments, and Negative healthcare environments. In contrast, the top four most common DIM categories for young people were: Withdrawal, Not being understood by people, Negative social environments and Frustration and anger. Figure 2(a) illustrates the frequency of each category and the marked differences in findings between both age groups. These differences were also supported by the frequency of proportions in the biological, psychological and social domains (see Figure 1). SIMs, however, were more similar between adults and young people. Both groups shared the same top four categories of Active self-management skills; Positive social environments; Enjoyable activities; and Positive self-talk and beliefs (Figure 2(b)), and there were similar proportions of SIMs across the biopsychosocial domains for adults and young people (Figure 1).

## Discussion

The four main themes of Engaging with the environment, In my body, My emotional health, and Activities and behaviours, and the underlying categories reflect a wide range of contributing factors to pain and pain relief. The themes summarise the key areas identified by people with pain, and these fit well with the biopsychosocial model of pain for both age groups. While themes were independently generated from analysis, they could be mapped onto the domains of biological (In my body), psychological (My emotional health) and social (Activities and behaviours; Engaging with the environment). The four derived themes provide an evidence-based interpretation for the seven more empirically formed original categories of the ‘Protectometer’. The large variety in categories reflects the breadth of DIMs and SIMs. It shows how individual factors contributing to pain and pain relief are, and how personalised pain management should be.

### A closer look at SIMs

The two main findings regarding SIMs were the similarity in SIM categories between adults and young people, and the psychosocial origin of most SIMs. Common SIM categories for both groups included Active self-management skills; Positive social environments; Enjoyable activities; and Positive self-talk and beliefs. These themes highlight the potential of integrative pain care approaches^25^ to provide treatments bolstering these common SIM areas. Indeed, interventions that take this biopsychosocial approach also tend to be those that induce improvements in pain and disability across age groups.^26–30^

That both age groups had the highest number of SIMs within the social domain was a finding of interest and relevance for future pain management approaches. The social and psychological domains of the biopsychosocial framework are often merged into a psychosocial domain, potentially undervaluing social factors.^31^ However, social factors such as feeling supported by friends,^32,33^ supportive relationships^34^ and leisure engagement^35^ are becoming increasing linked to improved health outcomes. Our study draws attention to the importance of the social domain, comprising of the physical environment (places), social environment (people), and meaningful occupations (activities) for people with pain. This finding also aligns with ‘social prescribing’ approaches, which are gaining momentum in pain management therapies.^36,37^ Social prescribing is a person-centred approach, which involves connecting people to community-based supports and activities to improve wellbeing.^38,39^ While addressing the social context can be seen as a cultural shift away from the medicalisation of health issues,^39^ it is appropriately placed within evidence-based biopsychosocial pain management care.^34^

### A closer look at DIMs

The differences in proportion of DIMs in the biological, psychological and social domains between adults and young people may be explained by several factors. Having less biologically based DIMs in young people may be related to having no or fewer co-morbid health conditions, a different understanding of pain mechanisms, or less time engaged with a biomedical healthcare model. It may also reflect the significance of psychosocial contributors to a young person’s experience of pain.^40^ This finding may raise the question, at what point along the developmental continuum do people begin to attribute their pain to more biological-based contributors? It could also be asked through what mechanisms (i.e. exposure to media, healthcare providers and/or family) do these perceptions change? Recent research has demonstrated that complex concepts involved in understanding pain are still developing in childhood, and are dependent on the knowledge, experience and cognitive development of the young person.^41,42^ Understanding more about how biopsychosocial factors contribute to pain from a young person’s perspective is likely to be helpful for tailored treatment approaches.

The most common types of DIMs for adults were in the Activity limitations and triggers category. Given the aversive nature of pain, it is unsurprising that activities linked to pain become signals of danger. Moreover, fear of those activities may persist beyond a period of time, and drive a cycle of ongoing disuse, distress, disability and pain.^43,44^ For young people, emotion-based DIM categories of Not being understood by people and Frustration and anger were more common. As chronic pain is often referred to as an ‘invisible illness’,^45^ and a condition associated with ageing,^46^ these DIM categories indicate young people may be left feeling with less credibility in their complaints, and poorly understood.

### DIMs, SIMs and the biopsychosocial model

It was noted during the analyses that some DIMs and SIMs could be coded into multiple domains of the biopsychosocial model. This observation reflects the ‘enactive model of pain’ as an extension of the biopsychosocial model.^47,48^ An enactive approach highlights the interaction between body, mind and environment, and removes boundaries from biopsychosocial domains.^48^ In an enactive approach, pain is considered an emergent process through a lived body that is inseparable from the world.^47^ This holistic approach fits well with some of the DIMs and SIMs in our study that described mutual influences across domains. Our data endorse current calls for more dynamic and bi-directional applications of the biopsychosocial model in treating chronic pain conditions.^49^

### Strengths and limitations

The inclusion of people with chronic pain in the content analysis was a strength of this study. While inclusive of young people and adult participants, a limitation was the large age range in both groups. This limited the ability to separate DIMs and SIMs into smaller age groups for analysis across developmental life stages. The relatively small number of young people in our sample (*n* = 19), due to COVID-19 pandemic restrictions, may limit generalisability of the findings in this age cohort. While not examined in this study, exploring the impact of specific conditions and pain types, and of culture and ethnicity on DIMs and SIMs should be of interest to the global pain community.

### Clinical significance

The overarching four themes provide insights into the most common types of DIMs and SIMs for adults and young people with pain. Our study adds to the growing body of research into tailoring pain science education^50^ by exploring age-specific DIMs and SIMs.

Specifically, for adults, this includes addressing perceived threats of activities, providing tools to manage sensory inputs, and enhancing social supports. For young people, tailoring interventions to address psychological and social domains, such as withdrawal from activities, recovery expectations and managing emotions, are important. Collectively, the commonly found SIMs in the social domain encourages clinicians to include social-based interventions as part of pain management therapies for both adults and young people.
